# The impact of COVID-19 on women’s mental health and coping during pregnancy and postpartum

**DOI:** 10.1038/s41598-026-37897-x

**Published:** 2026-02-10

**Authors:** Leslie C. M. Johnson, Rebecca Hong, Joi Henry, India Stevenson, Subasri Narasimhan

**Affiliations:** 1 Department of Family and Preventive Medicine, Emory School of Medicine, Emory University, 1518 Clifton Rd NE, Atlanta, GA 30322 USA; 2 Hubert Department of Global Health, Rollins School of Public Health, Emory University, Atlanta, GA USA; 3 Department of Behavioral, Social, and Health Education Sciences, Rollins School of Public Health, Emory University, Atlanta, GA USA

**Keywords:** Isolation, Loneliness, Depression, Anxiety, Qualitative, Maternal health, Patient education, Depression, Anxiety, Risk factors

## Abstract

The COVID-19 pandemic resulted in elevated rates of depression and anxiety among people during pregnancy, at rates higher than those observed in pregnant populations pre-pandemic. In this mixed methods study, 20 postpartum women were interviewed and administered depression and anxiety screeners to assess the mental health impacts of giving birth during the pandemic. Qualitative data were analyzed using a thematic approach, with subgroups comparisons to draw out differences in emotional, social, and psychological impacts, as well as coping mechanisms used. Descriptive statistics were used to describe the prevalence of anxiety and depressive symptoms. Most women held advanced degrees (85%), were married (95%), first-time parents (60%) and experienced a high-risk pregnancy (60%). Less than half (40%) of the women identified as a racial or ethnic minority. Women in this sample reported low rates of mild anxiety (30%) and depressive symptoms (15%). This study identified three themes related to mental health and coping during pregnancy and postpartum: isolation and loneliness as a result of COVID-19 disruptions, fear and anxiety resulting from simultaneous information overload and uncertainty surrounding COVID-19, and coping mechanisms to confront stress, anxiety, and loneliness. Community-based interventions to promote maternal mental health are needed to complement clinical support and resources.

## Introduction

Pregnant people are recognized as a vulnerable population requiring priority attention during public health emergencies such as the COVID-19 pandemic^1^. Pregnancy involves substantial biological, physiological, and psychosocial changes, and pregnant people are more likely than nonpregnant people to experience severe illness and hospitalization when infected with SARS-CoV-2, with associated risks for adverse obstetric outcomes^2,3^. During the pandemic, pregnant people also navigated evolving obstetric care guidelines and disruptions to health service delivery. Against this backdrop, the COVID-19 pandemic was associated with increased rates of depression and anxiety among pregnant populations compared with pre-pandemic levels^4,5^. Estimates indicate that during the COVID-19 pandemic, approximately one-third of pregnant individuals experienced clinically significant symptoms of depression and anxiety, with somewhat lower but still substantial prevalence reported among postpartum individuals (27.6% and 26.6%, respectively)^5–7^.

The COVID-19 pandemic intensified social isolation and fear of infection, adversely affecting mental health outcomes during pregnancy and after birth^8,9^. While the impact of COVID-19 on pregnant women is varied, a number of direct and indirect consequences have emerged. These include maternal complications such as preterm birth, gestational diabetes, preeclampsia, intrauterine growth restriction, miscarriage, and placental abruption; fetal complications including neonatal respiratory distress syndrome, sepsis, jaundice, hypoglycemia, and intraventricular hemorrhage; and adverse pregnancy outcomes such as preterm delivery, low birth weight, small for gestational age, and stillbirth^3,10^. For Black women in particular, the racial disparities in COVID-19 cases, hospitalizations, and death^11^ may have further contributed to stress and mistrust in the healthcare system^12^, particularly in the case of pre-existing experiences of racism when seeking obstetric care^13^. Aside from direct infection, the COVID-19 pandemic and mitigation policies also indirectly impact perinatal mental health through additional social stressors such as loss of employment, financial hardship, increased care obligations, or reduced social support due to cancellations^14^. These factors may contribute to an increase in mental health disorders among pregnant and postpartum women and their families^15^.

The COVID-19 pandemic produced immediate health care systems changes, with healthcare adaptations impacting the structure of pregnancy and birth care appointments and interpersonal interactions for a period of time, namely through changing visitor policies, reduced in-person care, and COVID testing requirements, to prevent the spread of COVID-19^16–19^. Previous survey data highlights how these care adaptations also impacted birth satisfaction, with the absence of birth partners and sufficient birth support leading to lower birth satisfaction^20^. Further research has linked lower birth satisfaction with a higher risk of postpartum anxiety, stress, and depressive symptoms, particularly among Black and Latina women^21,22^. These findings align with evidence demonstrating that social support is protective for perinatal women in reducing the likelihood of negative birth experiences, reducing risk of depression, and promoting mental wellbeing^23^. A lack of social support coupled with increased isolation throughout pregnancy and postpartum have been shown to contribute to a host of negative maternal health outcomes including stress, anxiety, and depression^24^.

For women who gave birth at the height of the COVID-19 pandemic, shelter-in-place orders, social distancing recommendations, and changes to hospital policies restricting the presence of support persons, coincided with a period when these individuals may rely heavily on social support systems^25,26^. Pregnant and postpartum women tended to decrease the size of their social circles during this time in an effort to reduce disease transmission, while disruptions to in-person support groups and classes made it difficult for mothers to establish new connections and community with other mothers^27^. More research is needed examining COVID-19’s impact on women’s mental, emotional, and social health during pregnancy and postpartum^28^, particularly among first-time parents and racial/ethnic minority groups, in order to identify appropriate approaches to support women’s psychosocial health in the perinatal period during future public health crises.

Through a mixed-methods study of women who were pregnant and gave birth in Georgia during the onset of the COVID-19 pandemic, our objective was to examine how the pandemic and health system changes impacting pregnancy and birth care affected women’s mental health and coping strategies. Secondarily, we sought to measure postpartum depression and anxiety symptoms in our sample.

## Methods

### Study design

This study employed a convergent mixed method phenomenological research design to explore women’s birth, delivery, and postpartum experiences during the COVID-19 pandemic. Participants completed a 60-minute semi-structured in-depth interview and a set of psychosocial questionnaires that included validated measures for depression and anxiety. Data collection took place between February and June 2022.

### Participants

Eligible participants were English-speaking adults (> 18 years old) who experienced a singleton pregnancy and delivery after March 2020, delivered and received their postpartum care in Georgia, and had access to a phone or computer to participate in the study activities. A total of 20 participants were recruited, purposively sampling women of color and first-time parents to capture a range of experiences. Individuals were recruited from the community through flyers posted in community spaces, pregnancy-related social media groups, and disseminated through the listserv of a nonpartisan, community-based organization that advocates for maternal and child health in Georgia.

### Procedures

Interested participants were screened to ensure that they met the study inclusion criteria. Eligible participants provided electronic written informed consent and were scheduled to complete an interview followed by a half an hour interviewer-administered questionnaire assessing participant’s psychosocial health through a series of validated scales.

An interview guide was developed to elicit birthing persons’ experiences seeking prenatal and postnatal care during the COVID-19 pandemic, with probes designed to encourage elaboration on how COVID-19 impacted their experiences seeking different forms of care and support. SN piloted and refined the interview guide based on discussion with the study team in debriefing the first interview. Changes to the guide included incorporating additional probes regarding the emotional aspect of participants’ experiences, particularly as it related to self-reported feelings of loneliness and unexpected changes in care. A trained interviewer, JH, conducted the remaining interviews. Interviews lasted 60–90 min and were audio-recorded.

All interview and questionnaire procedures were administered through Zoom. Study questionnaire data were collected and managed using Redcap’s electronic data capture tools hosted at Emory University^29^. REDCap (Research Electronic Data Capture) is a secure, web-based software platform designed to support data capture for research studies using an intuitive interface, validated data capture, audit trails to track manipulation, and automated data export. All study participants received $40 VISA gift cards after completing the interview and questionnaire. The study was approved by the Emory Institutional Review Board. Researchers adhered to the ethical standards set by the World Medical Association Declaration of Helsinki while performing the research^30^.

### Measures

Socio-demographic characteristics and information on birth outcomes and COVID-19 behaviors (e.g., social distancing, vaccination) were collected after participants provided informed consent through a brief (5 min) online, pre-interview survey. The psychosocial questionnaire included validated measures for depression and anxiety, described below. Participants were asked to indicate what affected their mental and physical well-being, with the ability to select any items that applied, including their birth experience, the COVID-19 pandemic, and other external factors, for which they could write-in a response. Lastly, participants were asked whether they had a high-risk pregnancy (e.g., being over 35 when pregnant, high blood pressure, previous pregnancy complications, etc.) where there was a higher risk of health problems before, during, or after the delivery for the mother or the fetus.

*Depression.* Depression was assessed using the Patient Health Questionnaire-9 (PHQ-9), a widely used and validated measure of depressive symptom severity in adult populations^31^. The PHQ-9 has been found to demonstrate substantial concordance with the Edinburgh Postnatal Depression Scale (EPDS) in identifying depression risk among postpartum women^32^. Items on the PHQ-9 closely follow the diagnostic criteria for Major Depression as defined in the *Diagnostic and Statistical Manual of Psychiatric Disorders* and are scored based on frequency of occurrence in the past two weeks from 0 (not at all) to 3 (nearly every day). The PHQ-9 total summative score ranges from 0 to 27, with cutoff scores of 5, 10, 15, and 20 indicating mild, moderate, moderately severe, and severe depressive symptoms, respectively. A final tenth item asks individuals to rate how difficult any indicated problems have made it for them to work, take care of things at home, or get al.ong with other people, with response options ranging from ‘not difficult at all’ to ‘extremely difficult.’

*Anxiety.* The General Anxiety Disorder scale (GAD-7) was administered to assess the presence and severity of generalized anxiety disorder among participants^33^. The GAD-7 has been validated for use in clinical and community settings^33,34^. Items are scored based on frequency of occurrence in the past two weeks from 0 (not at all) to 3 (nearly every day), with a total summative score of 0 to 21, with cutoff scores of 5, 10, and 15 indicating mild, moderate, and severe anxiety symptoms, respectively. An eighth item asks individuals to rate how difficult any indicated problems have made it for them to work, take care of things at home, or get al.ong with other people, with response options ranging from ‘not difficult at all’ to ‘extremely difficult.’

### Analysis

All interview data were transcribed verbatim using Happy Scribe, an automatic transcription and translation service^35^, and checked by a study team member for accuracy. Once transcribed and de-identified, the audio files were destroyed. Braun and Clark’s six-phase thematic analysis approach was used to identify salient issues related to mental health impact among women who were pregnant during the COVID-19 pandemic^36^. Three research staff members independently reviewed two transcripts to generate the initial codes and develop a codebook that was iteratively applied to transcripts until all segments were coded using the final codebook. Coding was conducted using Dedoose™, a cross-platform web-based application designed to analyze qualitative and mixed methods research^37^. Themes that provided interpretation of the data in response to the stated study aims were generated and data across those themes were then compared within participant sub-groups (e.g., age, race-ethnicity, first time parent status) to determine whether and how mental health and coping strategies varied among participants. Descriptive analyses were performed to determine the distribution of key sociodemographic and psychosocial variables using mean (SD, standard deviation) for continuous variables and frequency with percentage for categorical variables.

## Results

A full demographic breakdown of study participants is provided in Table 1. Participants in this study were predominantly Non-Hispanic White (60%), married (95%), held advanced degrees (85%), and were first-time parents (60%). The average age of participants was 34.6 years. At the time of data collection, just over half of participants were over a year postpartum and 60% self-reported having had a high-risk pregnancy.

**Table 1:** Demographic characteristics of participants.

Characteristic	Total (*n* = 20)
Age (years), Mean (SD)	34.6 (4.7)
	N (%)
Educational attainment	
Advanced degree	17 (85)
4-Year College degree	1 (5)
Some college	1 (5)
High School Diploma	1 (5)
Race/Ethnicity	
Non-Hispanic White	12 (60)
Non-Hispanic Black	6 (30)
Hispanic White	1 (5)
Multiracial	1 (5)
Marital status	
Married	19 (95)
Single	1 (5)
First-time parents	12 (60)
Months postpartum	
0–12	9 (45)
13–24	11 (55)
High-risk pregnancy	12 (60)
PHQ-9	
None	17 (85)
Mild	3 (15)
GAD-7	
None	14 (70)
Mild	6 (30)
Factors affecting physical and mental well-being	
Birth experience	6 (30)
COVID-19 pandemic	12 (60)
Other external factors	6 (30)

Six women (30%) reported mild anxiety symptoms, with GAD-7 scores in the sample ranging from 0 to 9. Despite generally low symptom severity, half of the participants indicated that their symptoms made it somewhat or very difficult to do work, take care of things at home, or get along with other people. The two most reported symptoms were feeling nervous, anxiety, or on edge (*n* = 10) and worrying too much about different things (*n* = 10).

While only three women (15%) reported having mild depressive symptoms, PHQ-9 scores in the sample ranged from 0 to 6. Despite generally low symptom severity, 11 participants (55%), including those with a PHQ-9 score of 2–4, reported that their symptoms made it somewhat or very difficult to do work, take care of things at home, or get along with other people. The majority of women reported having problems feeling tired or having little energy several days (*n* = 12, 60%), with a subset reporting having felt this symptom more than half of the days (*n* = 2, 10%), or nearly every day (*n* = 2, 10%) over the past 2 weeks. All the women who screened positive for mild depressive symptoms also screened positive for mild anxiety.

Additionally, 12 of 20 participants agreed that COVID-19 had an effect on their mental and/or physical wellbeing. Roughly a third of participants felt other external factors negatively impacted their mental and physical health, with identified factors including political climate in the U.S., international wars, climate change, transitions to parenthood as a working parent, body image, loss of a family member, and gun violence in schools.

The following sections detail three themes generated from the interview data related to mental health and coping during pregnancy and postpartum: isolation and loneliness as a result of COVID-19 disruptions, fear and anxiety resulting from simultaneous information overload and uncertainty surrounding COVID-19, and coping mechanisms to confront stress, anxiety, and loneliness.

### The COVID-19 pandemic interrupted social interactions and obstetric care resulting in isolation and feelings of loneliness

Disruptions to social interaction and community-building during pregnancy contributed to feelings of loneliness among participants. Efforts to minimize COVID-19 exposure limited opportunities to connect with friends, family, and broad support networks through events such as baby showers, resulting in a sense of detachment from usual social circles. Many participants described the loss of informal and structured avenues for maintaining social connection, and some turned to virtual forms of support as partial substitutes for in-person interaction.

I would say I felt lonely a lot of the time, and you don’t have the normal social gatherings. It was harder to see people just in general. It was incredibly lonely after having the baby because then we definitely weren’t seeing anyone. (White, 32 years old)

I think being pregnant during COVID-19 and being at home was very isolating. It was hard. I’m a very social person, very extroverted, so it felt challenging to not be able to be around people. But I have a lot of good friends that I was able to call and message and Facetime and Zoom with, especially those that had kids already and getting encouragement from them. (Asian, 33 years old)

A minority of participants did highlight how virtual contact was not wholly satisfying. The pandemic simultaneously isolated and increased fear among pregnant and postpartum people, with virtual interactions serving as imperfect substitutions.

More than half of patients, all of whom delivered by early 2021, reported that visitor restrictions for prenatal care visits made them feel lonely and deprived them of a major source of support during these clinical encounters. Several participants recounted feeling emotionally alone during critical points of their prenatal care, such as ultrasound screenings, and feeling disappointed that they could not share these moments with their partners. This was particularly true for women who experienced high-risk pregnancies, nearly all of whom reported feeling both physically and emotionally alone. At least one individual expressed that the benefits of having social support at clinical encounters outweighed the additional COVID-19 exposure risk, especially since spouses often shared the same living environment and took the same precautions to reduce exposure and spread of COVID-19. Participants with high-risk pregnancies also described their prenatal care experience as a period filled with anxiety because, at times, these individuals had to receive difficult news on their own. One participant who experienced an early miscarriage describes how painful it was to watch an ultrasound of her lost pregnancy while her partner could only support her over the phone. Another participant recounted the moment when she learned that her baby has a genetic disorder. She emphasized the emotional burden of taking in this information without her partner present and the lasting impact it had on her mental health:

But early on, after the genetic testing was done…we found out that she was going to have a high probability of having down syndrome, trisomy 21. So going through that by myself and seeing the genetic counselor, like, I wish he could have been there for that. I was reliving getting the phone call, sitting in meetings, and just thinking, ‘why do I have to retain all this information by myself when it’s emotional?’…and doing that alone was just a little more difficult. (White, 42 years old)

Another parent described how the clinical staff were not equipped to provide the necessary emotional and mental health support when recounting her experience having a miscarriage during the pandemic. She stated:

I will never forget the feeling of crying into an N95 by myself in a room because…an ultrasound tech was like, well, let me go find a provider to talk to you. So, she had to round up some random provider to talk to me about what the next steps were. (White, 36 years old)

Multiple participants expressed concern about hospital visitor restrictions forcing them to deliver in a hospital alone, however, this did not occur for any of the twenty who were interviewed; they were all permitted to have one visitor stay with them during the birth.

In the post-partum period, participants recovered and cared for their newborns in the same setting of isolation that had characterized the antenatal period. One participant described being anxious about COVID-19 exposure to her newborn and the lengths she and her partner took to protect their child:

I just would have loved to have been with all of the family and to have that community and pass the baby around or just kind of have that kind of connection. I feel like that was kind of really striking to me in terms of just the isolation. And we don’t take [my daughter] with us to go grocery shopping. We don’t take her with us to go anywhere. And so that feels unfortunate. (White, 36 years old)

The COVID-19 pandemic left participants alone in both the difficult and celebratory moments of pregnancy. The occurrence of pregnancy complications and loss, events that alone can negatively impact individuals’ mental and emotional health, were worsened by the restricted access to sources of support during earlier periods in the pandemic when stricter clinical visitation limits and social distancing measures were in place.

A sub-set of participants explicitly named that their decision to adhere closely to social isolation measures during the antenatal and postpartum periods was out of fear of COVID-19-related maternal complications. One participant severely restricted contact with her extended family members due to opposing views on the severity of COVID-19, and the fear that her family members were not taking proper precautions to prevent the spread of the virus to her and her newborn. The practice of social isolation could also be extreme for those who chose not to vaccinate and were afraid of contracting the virus. One participant, whose family chose not to vaccinate, had older children at home and started homeschooling them when in-person school returned.

### Simultaneous information overload and uncertainty surrounding COVID-19 led to fear and anxiety

More than half of study participants were pregnant in the early days of the pandemic, when information about COVID-19, the vaccine, and impacts on pregnancy was emerging rapidly with interpretations of this data varying among different sources of information and among the same sources over time. In this setting of uncertainty and simultaneous information overload, participants had to make important decisions, such as whether to get the COVID-19 vaccine, or how to adhere to social isolation measures. Participants sought health-related information from physicians, public health experts, and news outlets, as well as drawing on input from friends, neighbors, and the internet.

In the early days of the pandemic, participants felt that pregnant people were left out of the public health discourse about who was at high-risk for serious complications of COVID-19. They eventually learned that being pregnant put them at higher risk of infection, and also that infection could have very serious and deadly consequences for them.

But anything specific related to COVID pregnancy I think it was so new and so early that it was a I guess you would call us a high-risk group that wasn’t really being talked about as much as people who had respiratory issues or other autoimmune issues. It was, oh, and pregnant people, but we really don’t know what’s going to happen with them or what the impact will be. (Black, 43 years old)

At the beginning, it was just kind of like we - pregnant women weren’t even considered high-risk, and then we were at, a high-risk group. So, it kind of evolved. It’s like, oh, now you’re high risk. I was like, okay, well, I’m definitely not going to grocery stores and that sort of stuff. (White, 38 years old)

What began as fear of the unknown became rooted in reality as more news about pregnant people requiring hospitalization, or even dying, came to light. Participants also expressed fear of being separated from their newborns if they tested positive at delivery. Even considering that this cohort had heavy representation from highly educated public health workers, it was common for participants to struggle to digest data related to COVID-19 and make decisions about preventing infection. Participants frequently expressed being nervous about making the right decision when surrounded by the changing tides of information.

The constantly evolving information landscape was even difficult to navigate for the health professionals who were advising participants, which put further pressure on individuals to navigate different sources of sometimes contradictory advice. One participant signed up to receive the vaccine when it was first released and said that the pharmacy administering it tried to deny her upon learning she was pregnant. She describes how she had to press hard to convince the pharmacist to give her the vaccine:

She came with the shot and was holding it and I remember it so vividly, she goes, ‘I don’t know what this can do to your baby and so I’m not sure if you should get vaccinated or not. So if something happens to your baby, then I can’t be held responsible and I want you to make sure that you actually want to do this.’ And I was sitting there and I was like, yeah - and I literally had the [American College of Obstetrics and Gynecology] report printed out and everything. I was like ready to go because I figured they were going to be a pain…She was like, ‘you’re the first [pregnant] one…I could be liable if your baby dies.’ (Hispanic, 33 years old).

In another example, one individual explains how her obstetrician changes his mind about vaccination during her prenatal care:

I talked to my [obstetrician] about [vaccination] and because he didn’t know anything about it, he was like, I don’t want you to take it…But I think he must have learned more about it because when I started to see the [maternal fetal medicine specialist] and I asked him about it again, he was like, yeah, you probably should take it. (Black, 38 years old)

Access to abundant information was described as having both negative and positive impacts for pregnant women. There was so much to take in that in some cases it was reported to cause confusion, create discord between patients and healthcare workers, and cause anxiety for pregnant women who had to make important decisions about their health. At the same time, information could also be empowering if they trusted the source and data provided.

### Using multiple coping mechanisms to confront stress, anxiety, and loneliness

**Fig. 1:**
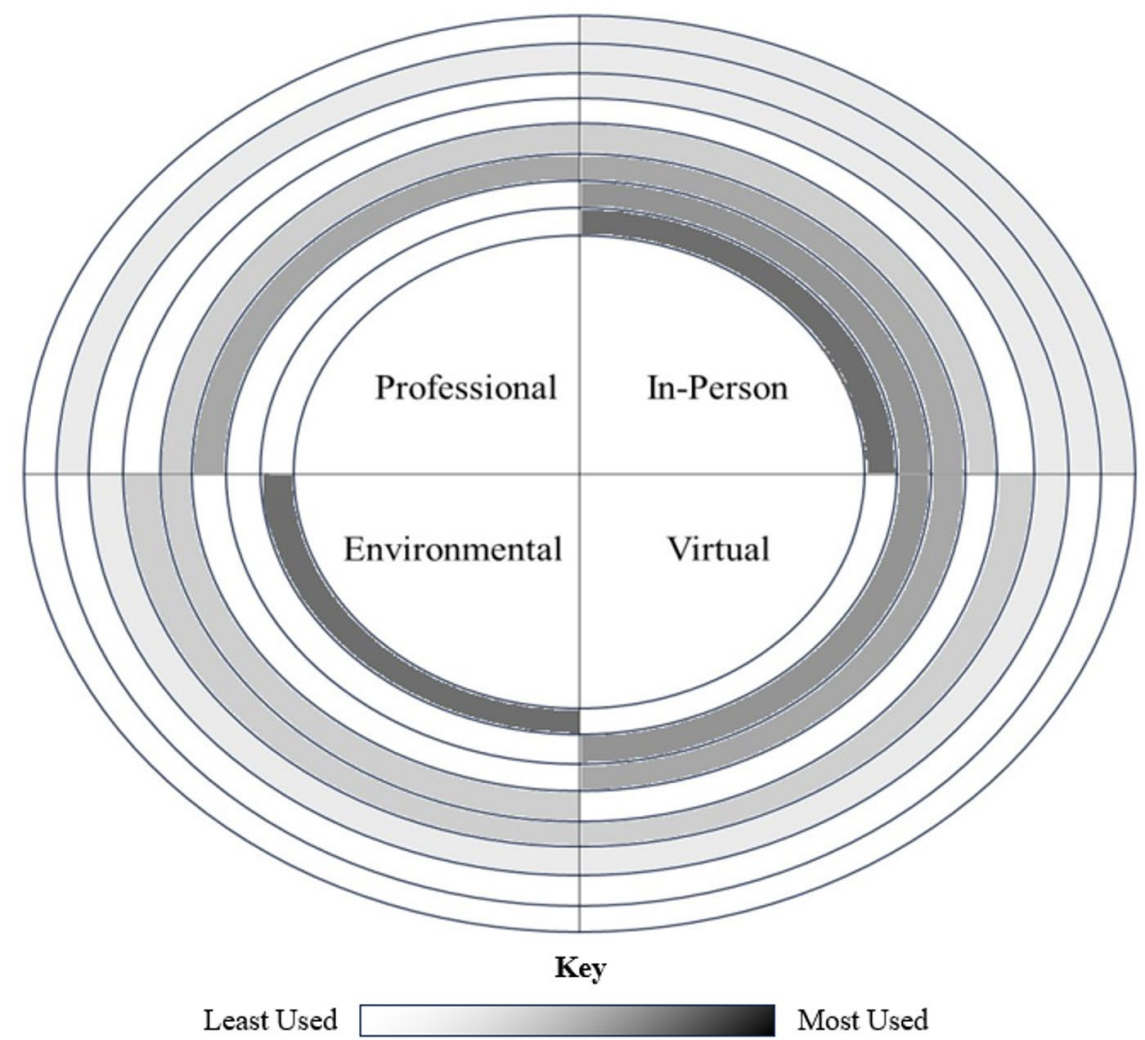
Frequency and combinations of mechanisms used to cope with stress, anxiety, and loneliness. In this figure depicts the combination of coping mechanisms used by study participants to cope with feelings of stress, anxiety, and loneliness. Reported strategies were clustered into four categories: (1) treatment by mental health professionals (i.e., Mental Health Treatment), (2) engaging in in-person interactions with people in one’s social circle (i.e., In-Person), (3) engaging in on-line interactions with people within and beyond one’s social circle (i.e., Virtual), and (4) spending time outdoors to experience a shift in environment and take comfort in natural surroundings (i.e., Environmental). Participants varied in their coping approaches, with some relying on a single strategy and others using multiple strategies across categories. There were eight reported combinations of the four types of coping mechanisms, displayed by the presence of one or more highlighted quadrant segments in each ring. Each rung illustrates which coping mechanisms co-occurred and how frequently they were used, with the most commonly reported combinations appearing in darker shading and positioned closer to the center, and less frequently reported combinations extending outward. For example, the most common pattern involved a combination of environmental and in-person coping mechanisms, whereas fewer participants reported relying solely on in-person coping mechanisms.

Approximately a quarter of participants (all first-time parents) shared that they accessed professional mental health resources during their pregnancies. Only one person self-disclosed that she had been diagnosed with generalized anxiety in pregnancy and reported accessing mental health services in the postpartum period. She expressed that the main barrier to getting mental health care at that time was the availability of mental health providers, stating “The demand for therapists is so high that just the wait times to get an appointment are huge.” Women developed their own support networks and coping mechanisms, often combining different methods in order to tend to their mental health and break up periods of isolation (see Fig. 1 for a depiction of how different combinations of coping strategies were used in this cohort). Participants described relying on virtual support groups, doula services, and prenatal classes throughout their pregnancies. They used outdoor spaces for recreation and to find a respite from isolation and stress inside the home. Many also cited their partners as a source of support during their pregnancy. Finally, women found support in their friends, extended family, and neighbors, often seeking out pregnant peers in these networks for solidarity.

Online engagement was the most common way participants reported accessing support. Half of the participants mentioned that they relied on virtual support services during their pregnancies, such as Facebook mom groups, with virtual platforms also serving as a way to celebrate pregnancy.

I think outside of the health care setting, I would encourage any pregnant woman, even if you’re in the middle of a pandemic, still do those things that you wanted to do. Even if it’s going to be out of my smaller scale, if you wanted to have a gender reveal, do a virtual gender reveal, which is what I did…because it’s important to mark it. I think it just gives you strength sometimes when this can be so stressful. [Pregnancy] is definitely a physical experience, but I think the mental and the emotional and the social is critical. (Black, 33 years old)

Through technology, pregnant women could find relief from some of the loss and stress associated with isolation. On the other hand, more than half of women took to outdoor spaces for recreation and stress relief. They took up hobbies such as hiking and gardening as these were considered safe activities that bore low risk of infection.

When detailing how they coped with the stress and anxiety of being pregnant during the pandemic, women described how they drew on broad networks for support. Most credited their partners as a vital source of support, with two using their husbands as a stand in for a doula, but they also named family members, neighbors, friends, coworkers, and church members as sources of comfort. These individuals shared parenting tips over text messages, helped run errands, joined them for walks, dropped off food when checking in on them, among other things.

Two other girlfriends were pregnant with me during my first pregnancy. And we talk every day, all day on the text about how all the babies, all the spouses, how everybody is doing all the time. And they are a constant support network. (White, 34 years old)

This was in contrast to one participant who expressed not having a strong support network during pregnancy. When asked what support she felt was missing, she replied:

I wanted to be building a village, like building that community, and that didn’t get to happen. So, there was no one to call or text to be like, just with whatever with random questions or to commiserate. (White, 30 years old)

Though this individual participated in virtual support groups, she felt that speaking with strangers on the internet was not an equivalent stand in, demonstrating the limits of virtual platforms.

## Discussion

This paper sought to examine how the COVID-19 pandemic and health system changes during the pandemic affected pregnant women’s mental health and coping strategies. We found that visitor restrictions for prenatal visits were particularly distressing and that women classified with high-risk pregnancies were more likely to report struggling with emotional isolation. The healthcare system could not provide an adequate substitute for the support desired during the stressful prenatal period. Further, their loneliness was not confined to clinical encounters; participants practicing social distancing felt isolated from friends, family, and other sources of support, and they missed out on opportunities to build and maintain community during pregnancy. Consistent with emerging work on resilience during the COVID-19 pandemic^38,39^, the erosion of interpersonal and partner support represented a key stressor for maternal mental health, while access to supportive relationships functioned as an important buffer against distress. Importantly, these findings suggest that needs for connection, predictability, and emotional safety during pregnancy are foundational, rather than contingent on individual education, health literacy, or professional background. Participants reported that the health system vacillated between sharing little and then so much information about the dangers of COVID-19 in respect to pregnancy, causing confusion and fear for some pregnant women. To cope with feelings of loneliness, fear, and sadness, participants drew on a range of coping strategies that exist outside of the health system, such as peer support and self-directed practices. The pandemic shaped access to and reliance on these resources, underscoring the role of informal social support and adaptive coping as resilience mechanisms during periods of acute disruption.

Secondarily, we sought to measure depression and anxiety symptoms in our postpartum sample. Documented rates of depression and anxiety among pregnant and postpartum women during the pandemic vary widely, with prevalence rates ranging from 2 to 64% and 22.7–60%, respectively^6,7,40,41^. Rates of reported depressive and anxiety symptoms in this cohort (15% and 30%, respectively) are lower than rates documented in other samples of postpartum women during the COVID-19 pandemic^42^. However, most of those studies conducted mental health screenings one week to three months after delivery whereas women in this study were 3–24 months postpartum. Despite reporting fewer symptoms of anxiety and depression, study participants reported that their symptoms made it somewhat or very difficult to do work, take care of things at home, or get al.ong with other people; suggesting that functional impairment may persist even when symptoms severity is lower, and that resilience does not preclude ongoing psychosocial strain.

While higher socioeconomic status and education could be protective for maternal mental health in a normal context^43^, during the COVID-19 pandemic, there is some data demonstrating the contrary^44^. Similarly, this study had high representation of individuals with advanced education; only one participant’s highest level of education was a GED, while the majority had a graduate-level degree and worked in the public health sector. Highly educated individuals in our sample were able to maintain employment during the pandemic and work from home. They also had access to the latest public health guidance regarding COVID-19 and the means to access professional mental health services. Our interviews support that even among this group of individuals, who had relative financial stability and access to health resources, pregnant women in the pandemic experienced feelings of isolation, stress, and anxiety, reinforcing evidence that social support and relational connectedness, rather than material resources alone, are central to psychological resilience during widespread crises^8,38^. From an equity perspective, these findings suggest that system-level disruptions can undermine resilience even in comparatively advantaged populations, highlighting the likelihood of amplified impacts among those with fewer resources or less institutional trust.

Our study suggests that individuals with high-risk pregnancies were more likely to report feelings of isolation and anxiety during the prenatal period, consistent with prior evidence identifying high-risk pregnancy as a risk factor for depression and anxiety^45^. Our interviews offer one explanation for this association in the context of the early COVID-19 pandemic. Among participants who were pregnant in 2020, visitor restriction policies during prenatal visits often required women to process complex or distressing clinical information without partner support. Concurrent social distancing measures further limited access to emotional support beyond the immediate household. Some participants attempted to mitigate this isolation by recording visits or using videoconferencing to include partners, although such efforts were sometimes restricted, particularly during ultrasound appointments. These findings align with prior work demonstrating that partner involvement and perceived social support are critical protective factors for maternal mental health and resilience during periods of heightened prenatal stress^38,39^. Together, these experiences point to the importance of health systems that are prepared to operate under uncertainty by prioritizing transparent communication, clearly articulating what is known and unknown, and engaging patients as partners in decision making. These findings highlight important lessons for future public health emergencies that necessitate infection control measures. In such contexts, health systems should consider flexible policies that preserve partner involvement through virtual participation or other low-risk accommodations to support maternal mental health while maintaining patient safety.

Another concept that was reiterated in our study was the relationship between information and feelings of fear and anxiety. Other studies have demonstrated that too little information about COVID-19 could lead to anxiety and depression, while too much information about the risks and mortality rate could also negatively affect mental health^4^. Pregnancy is a state of immunosuppression and pregnant people are both more susceptible to respiratory illness and can develop more serious symptoms as a result. In a pandemic of a novel respiratory virus, pregnant people should be a priority for the development of care recommendations. Our findings suggest that beyond the content of information itself, the consistency, transparency, and rationale behind changing guidance are critical for supporting trust and psychological safety. In terms of information overload, it is important to acknowledge that pregnant individuals are consuming large amounts of information via the news and social media that can impact mental health.

Participants in this study dealt with isolation and negative emotions in ways consistent with what has been reported in the literature. One large cross-sectional study of women in the US showed that talking with others, going outdoors, and more screen time were the top three most utilized coping mechanisms, and that relying on the first two methods were significantly correlated with reduced anxious and depressed symptoms^46^. Mirroring findings from other studies^47–49^, women tried to combat loneliness and receive pregnancy support from spouses and through accessing virtual support groups and socially distanced in-person visits, reflecting adaptive coping strategies that have been identified as key components of resilience in perinatal populations during the COVID-19 pandemic^8,39^. These findings underscore the need for perinatal care systems that proactively support social connection, flexibility, and shared problem solving, particularly during periods of disruption, as a pathway to strengthening resilience and advancing equity in perinatal mental health.

This study was strengthened by its use of mixed methods and inclusion of women who were pregnant at varying times at the onset and throughout the on-going COVID-19 pandemic. As a result, our findings reflect experiences spanning multiple phases of the pandemic, from its onset in March 2020 through later periods characterized by the Delta and Omicron variants, during which public health restrictions and health system disruptions varied over time. However, we did not purposively sample participants based on the timing of pregnancy or delivery relative to specific phases of the pandemic or hospital-level restrictions, which varied across settings and over time. Consequently, we were unable to systematically examine how differences in visitor policies, care practices, or restriction intensity shaped experiences during distinct pandemic phases. We chose to verbally administer the psychosocial questionnaire, including depression and anxiety measures, to reduce barriers to participation, however, this approach may have resulted in response bias. Because the questionnaire was administered following the interview with the same study team member, a good level of rapport had already been established to offset the potential for response bias. Additionally, the cohort sampled heavily from individuals in the public health field with high levels of education and may not reflect the experience of the general population. Although the Edinburgh Postnatal Depression Scale is commonly used in postpartum populations in the year following delivery, we selected the PHQ-9 to allow consistent assessment across a broader postpartum time frame; prior evidence demonstrating substantial concordance between the PHQ-9 and EPDS in postpartum populations suggests this choice is unlikely to have meaningfully altered depression risk classification^32^.

## Conclusion

Although the acute phase of the COVID-19 pandemic has passed, its effects have underscored how quickly social disruption, uncertainty, and constrained health care access can exacerbate mental health vulnerability during pregnancy and the postpartum period. The elevated burden of depression and anxiety observed during the pandemic should not be viewed as an anomaly, but as evidence of persistent gaps in perinatal mental health support that remain under routine conditions. Strengthening community-based mental health interventions alongside clinical care is therefore an urgent priority, particularly for pregnant and postpartum persons who face barriers to consistent health care access. Proactive, inclusive strategies that extend beyond emergency response are needed to ensure timely, equitable mental health support across the perinatal continuum and to build resilience ahead of future public health disruptions.

## Data Availability

The datasets generated during and analyzed during the current study are available from the corresponding author on reasonable request.
